# Search for atoxic cereals: a single blind, cross-over study on the safety of a single dose of Triticum monococcum, in patients with celiac disease

**DOI:** 10.1186/1471-230X-13-92

**Published:** 2013-05-24

**Authors:** Barbara Zanini, Beatrice Petroboni, Tarcisio Not, Nicola Di Toro, Vincenzo Villanacci, Francesco Lanzarotto, Norberto Pogna, Chiara Ricci, Alberto Lanzini

**Affiliations:** 1 Gastroenterology Unit, University and Spedali Civili of Brescia, Brescia, Italy; 2 Paediatric Gastroenterology, Burlo-Garofolo Hospital and University of Trieste, Trieste, Italy; 3 Histopathology Unit, University and Spedali Civili of Brescia, Brescia, Italy; 4 Research Unit for Cereals Development, CRA, Rome, Italy

**Keywords:** Triticum monococcum, Intestinal permeability, Toxicity, Celiac disease

## Abstract

**Background:**

Cereals of baking quality with absent or reduced toxicity are actively sought as alternative therapy to a gluten-free diet (GFD) for patients with coeliac disease (CD). *Triticum monococcum*, an ancient wheat, is a potential candidate having no toxicity in in-vitro and ex-vivo studies. The aim of our study was to investigate on the safety of administration of a single dose of gluten of *Tm* in patients with CD on GFD.

**Methods:**

We performed a single blind, cross-over study involving 12 CD patients who had been on a GFD for at least 12 months, challenged on day 0, 14 and 28 with a single fixed dose of 2.5 grams of the following (random order): Tm, rice (as reference atoxic protein) and Amygluten (as reference toxic protein) dispersed in a gluten-free pudding. The primary end-point of the study was the change in intestinal permeability, as assessed by changes in the urinary lactulose/rhamnose ratio (L/R ratio) measured by High Pressure Liquid Chromatography. We also assessed the occurrence of adverse gastrointestinal events, graded for intensity and duration according to the WHO scale. Variables were expressed as mean ± SD; paired t-test and χ^2^ test were used as appropriate.

**Results:**

The urinary L/R ratio did not change significantly upon challenge with the 3 cereals, and was 0.055 ± 0.026 for *Tm Vs* 0.058 ± 0.035 for rice (p = 0.6736) and Vs 0.063 ± 0.054 with Amygluten (p = 0.6071). Adverse gastrointestinal events were 8 for Tm, Vs 11 for rice (p = 0.6321) and Vs 31 for Amygluten p = 0.0016), and, in all cases events were graded as “mild” or “moderate” with TM and rice, and as “severe” or “disabling” in 4 cases during Amygluten.

**Conclusions:**

No definite conclusion can be drawn on the safety of Tm, based on no change in urinary L/R because even Amygluten, a toxic wheat protein, did not cause a significant change in urinary L/R indicating low sensitivity of this methodology in studies on acute toxicity. *Tm* was, however, well tolerated by all patients providing the rationale for further investigation on the safety of this cereal for CD patients.

**Trial registration:**

EudraCT-AIFA n2008-000697-20

## Background

Lifelong adherence to a strict gluten free diet (GFD) is at present the only treatment for patients with celiac disease (CD) [1] to reduce morbidity and mortality. Compliance to GFD is however difficult and affects the quality of life of patients because, besides economic and social factors [2,3], it involves the consumption of poorly palatable unleavened bakery products. This is the reason why alternative strategies are actively sought [4], which include the search for baking quality wheat that does not contain toxic gluten. This strategy takes advantages of the notion that there is natural variation in grain toxicity [5,6], and the old diploid grass-like species of Triticum genus are potential candidates as grains with reduced or absent toxicity. In particular *Triticum monococcum* (TM) has been shown to contain a low number of stimulatory epitopes of Tcell lines obtained from small intestinal biopsies on CD patients [6], and to lack the genes encoding the immunodominant 33 mer fragment [5]. Furthermore, the presence of a “protective” peptide similar to the 10-mer peptide (QQPQDAVQPF) of Durum wheat [7] has been detected in Tm [8].

Preliminary in-vitro and ex-vivo studies have provided encouraging results. Absent in vitro toxicity has been reported from in vitro studies, where Tm was unable to agglutinate K562(S) cells [9] and had no effect on NO and TGII expression in Caco-2/TC7 cells [10]. Absent toxicity of Tm has been reported by Pizzuti et al. [11] in a study ex vivo showing no morphological changes in duodenal biopsies cultured with peptic-tryptic digest of Tm gliadin. Taken together, the studies reported above suggest a favourable safety profile of Tm for CD patients and provide the rationale for testing Tm administration for toxicity in CD patients. It is however noteworthy that, in contrast with previous studies, in vitro toxicity of Tm has been recently reported by Gianfrani et al. [12]; such information was not available when we planned our study.

The aim of our study was to assess the safety of Tm administration by challenging CD patients complying with a GFD with a single 2.5 g protein extract of Tm by comparison, in random order, with that of an atoxic protein extract of rice, and with that of a toxic gluten, Amygluten. We measured changes in urinary recovery of lactulose and rhamnose (L/R) as an experimental biomarker of intestinal permeability [13].

## Methods

We selected 12 consecutive CD patients on GFD for at least one year, at follow-up in our Celiac Clinic, and meeting the following selection criteria: strict compliance with the GFD, absence of symptoms, reconstitution of villous structure and negative tissue transglutaminase (t-TG) and/or antiendomysial (AMA) antibodies during GFD. Compliance with the GFD was assessed as previously described [14] using a 4 point Likert scale that includes no dietary indiscretions (score 1), 1 serving with gluten per month (score 2), < 4 servings per month (score 3) or = > 4 servings per month (score 4). We also selected 7 CD patients freshly diagnosed with villous atrophy and positive CD related serology and on a gluten containing diet. Twelve asymptomatic healthy subjects selected among the health care professionals in our Institution volunteered in the study as normal controls.

CD patients on GFD entered a single blind cross over study involving challenge with 3 proteins (random order): rice (MyProtein, Cent Ltd, Northwick, UK) as atoxic control, pure gluten (Amygluten, Tereos Syral, Marckolsheim, France) as toxic control, and Tm (*Triticum monococcum* ssp *monococcum*, cultivar “Monlis”, CRA, Rome, Italy) as investigational protein. Challenge with different proteins was carried out on 3 separate occasions on day 0, 14 and 28. The primary endpoints were the effect of challenge on the urinary recovery L/R ratio as a measure of intestinal permeability, and the effect on symptoms. Patients were instructed to report any symptom experienced during the challenge, which was graded for severity according to the WHO toxicity grading scale as mild (grade 1), moderate (grade 2), severe (grade 3) and life threatening (grade 4) [15]. We used a 2.5 g protein dose for the challenge, corresponding approximately to one slice of bread and to the dose used by others [16-18] for “proof of the concept” challenge studies.

The timetable in the study was as follows: fasted patients were admitted to a day-case Unit, asked to empty the urinary bladder and immediately after they were asked:

t 0 to eat a gluten free pudding (BiAglut VAN, Heinz, Italy) with dispersed 2.5 g cereal protein

t 2 h to drink a solution of 5 g lactulose + 1 g rhamnose in 60 ml water

t 2-7 h to collect urine and to record symptoms

At t 7 h urine volume was measured, a sample was retained, frozen and stored until analysis. Urinary samples were analyzed by using HPLC [19] for L and R concentration and for calculation of the L/R ratio in one batch for each patient. The normal urinary L/R, for our laboratory, calculated in 40 healthy controls is 0.045. All analyses were carried out at the Burlo-Garofolo Paediatric Hospital in Trieste (Italy) under the supervision of one of the authors (T.N.).

The same protocol used for CD patients on GFD challenged with cereal proteins was also adopted, with the exclusion of protein challenge, to measure intestinal permeability in 7 CD patients on gluten containing diet and in 12 healthy controls. Five CD patients on GFD and 5 healthy controls were also studied with the same protocol on 2 occasions, one day apart, to test for reproducibility of results.

Results were expressed as mean ± SD. Paired or unpaired t-test and χ^2^ test were used as appropriate to compare continuous and categorical variables. The statistical analysis was carried out using GraphPad Prism 5 statistical package (GraphPad Software, San Diego, Ca, USA). The study was approved by the Ethics Committee of Spedali Civili of Brescia on February 5th, 2008 and was given the number n2008-000697-20 in our national registry of clinical trials (EudraCT-AIFA). Patients and controls signed a written informed consent to the study.

## Results

Anthropometric and clinical characteristics of the subjects enrolled are reported in Table 1. All 12 CD patients had normal duodenal mucosa, and serology was negative during GFD in all but 1 patient who was t-TG negative and weakly positive at EMA testing. All seven CD patients on gluten containing diet had similar characteristics to patients on GFD, and all had duodenal atrophy and tested positive at serology. Mean age was lower and M/F was similar in the 12 healthy subjects as in CD patients.

**Table 1:** Characteristics of celiac patients on gluten free diet (GFD) (panel A), on gluten containing diet (panel B), and of healthy controls (panel C)

	**Marsh**						**t-TG**	
	**Age**	**Sex**	**BMI**	**Baseline**	**GFD**	**Baseline**	**GFD**	**GFD**
	**(years)**					**(U/mL)**		**(years)**
**A**	53	M	23.8	3B	2	5/5	6/16	5
	46	F	19.5	3C	2	8/7	1/7	18
	50	M	26.5	3C	2	3/8	1/7	4
	48	M	25.4	3A	2	15/8	4/9	4
	35	F	22.7	3A	2	23/7	7/9	2
	42	M	21.6	3C	2	9/7	2/16	2
	35	f	18.4	3B	2	99/5	8/9	4
	62	F	21.5	3C	2	22/7	7/16	2
	31	F	26.6	3A	2	19/16	3/16	1
	59	F	24.7	3B	0	12/9	5/16	2
	32	F	18.4	3C	2	12/9	2/4	6
	41	F	25.03	3C	2	107/9	13/16	2
X	44.5		22.8					
SD	10.3		3.0					
**B**	52	M	26.0	3C		159/16	-	-
	37	F	20.7	3C	-	96/16	-	-
	42	F	21.5	3A	-	29/16	-	-
	16	F	18.0	3A	-	22/16	-	-
	42	M	27.4	3B	-	153/16	-	-
	41	F	25.4	3C	-	100/9	-	-
	36	F	24.2	3B	-	157/16	-	-
X	40.9		23.3					
SD	14.3		3.4					
**C**	36	F	21.7					
	25	M	23.1	-	-	-	-	-
	63	M	26.4	-	-	-	-	-
	42	M	23.4	-	-	-	-	-
	26	F	18.9	-	-	-	-	-
	25	F	21.1	-	-	-	-	-
	28	F	24.5	-	-	-	-	-
	36	F	19.9	-	-	-	-	-
	26	M	20.8	-	-	-	-	-
	26	F	20.3	-	-	-	-	-
	25	M	21.7	-	-	-	-	-
	39	M	30.7	-	-	-	-	-
X*	33.1		22.7					
SD	11.3		3.3					

* : p = 0.0168 by comparison with panel A.

### Validation study

**Figure 1:**
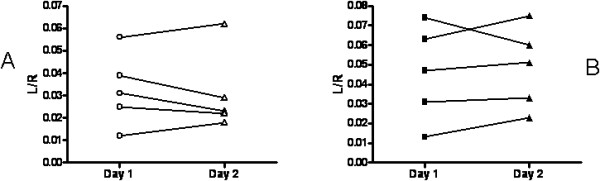
Reproducibility on 2 successive days of urinary lactulose/rhamnose ratio (L/R) as an index of intestinal permeability in (A) healthy controls and (B) celiac patients.

Urinary L/R was higher in CD patients on gluten containing diet (0.078 ± 0.022) than in controls (0.052 ± 0.031, p = 0.0345) and in patients on GFD (0.058 ± 0.034, p = 0.1852). Five control subjects and 5 CD patients entered the reproducibility study. Mean value of L/R was 0.046 ± 0.024 Vs 0.048 ± 0.021 (p = 0.5746) and was 0.033 ± 0.016 Vs 0.031 ± 0.018 (p = 0.6228) on day1 Vs day 2 in CD patients and control subjects, respectively, and coefficient of variation of measurements was 5.4% and 5.3% (Figure 1).

Urinary recovery of R ranged between 92% and 98% in urinary samples of the 31 subjects cumulatively entering the study.

### Cereal challenge

**Figure 2:**
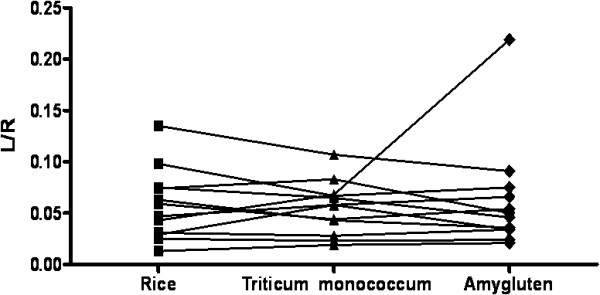
Effect of acute challenge with 2.5 g of rice protein, Triticum monococcum protein and Amygluten on changes of intestinal permeability as measured by urinary lactulose/rhamnose ratio (L/R) ratio in celiac patients on gluten free diet.

Results of urinary L/R in the 12 CD patients on GFD challenged with 3 cereals are shown in Figure 2. There was no consistent trend for L/R ratio to change during acute challenge with Tm, Amygluten or rice, and mean values were 0.055 ± 0.03 for Tm Vs 0.058 ± 0.035 for rice (p = 0.6736) and Vs 0.063 ± 0.02 for Amygluten (p = 0.6071).

The effect of cereal challenge on symptoms is shown in Table 2. Eleven and 8 adverse gastrointestinal events were reported during challenge with rice and with Tm (p = 0.6321), respectively, and 31 events were reported during Amygluten, a significantly (p = 0.0016) higher value than for the other 2 cereals. Severity of adverse events was graded as mild or moderate with rice and Tm, and as “severe” or “disabling” in 4 cases during Amygluten.

**Table 2:** Number of adverse events reported during challenge with the 3 cereals studied

	**Rice**	**Triticum monococcum**	**Amygluten**
**Symptoms**	**n**	**n**	**n**
Abdominal pain	1	2	7
Bloating	2	1	11
Constipation	-	-	-
Diarrhoea	2	-	-
Flatus	-	-	-
Disgeneusia	1	-	2
Nausea	5	5	9
Vomiting	-	-	2
Heartburn	-	-	-
**Total n**	11	8	31

## Discussion

The main objective of our study was to assess the effect of challenge with Tm in CD patients on GDF using urinary L/R ratio as a method to measure changes in intestinal permeability in order to test in vivo safety and toxicity of a single low dose of Tm. Our results show that urinary L/R ratio was unchanged during Tm challenge in comparison with the results obtained with rice, an atoxic cereal for CD patients. This potentially interesting observation is however of limited interest because, as in Tm, even challenge with the toxic reference protein Amygluten caused no significant change in the urinary L/R ratio in relation to that measured during rice challenge. The reason for this lack of effect of Amygluten in uncertain. Our preliminary validation studies indicating high reproducibility of results for urinary L/R recovery both in celiacs and in healthy controls, and the ability of the test to discriminate healthy controls from CD patients on gluten containing diet, support the validity of methodology used for measurements. On the other hand, the lack of effect observed during challenge with toxic Amygluten indicates inadequacy of the experimental conditions for testing the working hypothesis. The most likely explanation is that the protein dose used for challenge, 2.5 g as a single dose, may be too low to cause the alteration of intestinal permeability that was reported by Greco et al. [13] which occured using 50 g protein challenge. Alternatively, the timing of urinary collection may be inadequate for detecting changes in the L/R ratio. Whatever the case, the methodology we used was clearly not sensitive enough to achieve the aims of our study.

Though results on urinary L/R ratio were disappointing, results on symptoms reported by patients during the challenge provided a clear-cut response, indicating that a single low dose of Tm is well tolerated by CD patients. Symptom incidence with Tm was similar to that observed during challenge with rice, the atoxic cereal, and symptoms were in all cases mild. In contrast, incidence of symptoms was 3 times higher during Amygluten than during Tm and rice challenge, indicating that the dose used for challenge was large enough to cause symptoms in case of toxicity. This clinical finding is in keeping with previous in vitro and ex-vivo observations suggesting no toxicity of Tm for CD patients, although we are well aware that our finding on symptoms cannot be taken as evidence of lack of toxicity of Tm.

## Conclusions

In conclusion our study indicates that a protocol involving short-term challenge with a single low dose of cereal protein using urinary L/R recovery is not sensitive enough to discriminate the effect of toxic and atoxic cereals on intestinal permeability to sugars. As a consequence, no conclusion can be drawn on the safety of acute administration of Tm on CD patients. However, the lack of side effects reported by patients during challenge with Tm encourages to further explore the characteristic of this cereal as a potentially harmless wheat for CD patients, or as a cereal that may be tolerable for patients who are not celiacs but do not tolerate wheat based products because of gluten sensitivity.

## Abbreviations

CD: Celiac disease; GFD: Gluten free diet; R: Rhamnose; L: Lactulose; Tm: Triticum monococcum; HPLC: High Pressure Liquid Chromatography.

## Competing interest

A.L. financial support to the study from “Fondazione Antica Terra”.

All other authors declare that they have no competing interest.

## Authors’ contributions

BZ: participated in conception and design of the study, coordinated the challenge procedure and carried out the analysis and interpretation of data. BP: coordinated the challenge procedure, participated in analysis and interpretation of data. TN: carried out the analysis of urinary rhamnose and lactulose. NDT: carried out the analysis of urinary rhamnose and lactulose. VV: revised biopsies of patients involved. FL: performed endoscopic biopsies, participated in analysis and interpretation of data. NP: provided relevant background information and participated in analysis and interpretation of data. CR: participated in analysis and interpretation of data. AL conceived and designed the study, participated in analysis and interpretation of data, wrote and finalized the manuscript. All authors read and approved the final manuscript.

## Pre-publication history

The pre-publication history for this paper can be accessed here:

http://www.biomedcentral.com/1471-230X/13/92/prepub
